# Restarting Elective Orthopaedic Surgery During the COVID-19 Pandemic: Lessons Learned

**DOI:** 10.7759/cureus.16343

**Published:** 2021-07-12

**Authors:** Anuhya Vusirikala, Marwan Saleh, Edward Laurent, Tessa del Castillo, Ranjith R Kuzhupilly, Amr Fahmy, Dimitrios Tsekes

**Affiliations:** 1 Trauma and Orthopaedics, Basildon University Hospital, Mid and South Essex NHS Foundation Trust, Basildon, GBR; 2 Spine Surgery, Basildon University Hospital, Mid and South Essex NHS Foundation Trust, Basildon, GBR

**Keywords:** protocol review, elective orthopaedic surgery, postoperative complication, surgical pathway, covid-19

## Abstract

Introduction

Coronavirus disease 2019 (COVID-19) resulted in postponing non-emergency elective surgeries beginning in April 2020. Our hospital successfully restarted elective orthopaedic surgery during the pandemic to help improve the quality of life of patients with chronic disabilities.

This study describes the development of local protocols and pathways to allow for a safe restart of elective orthopaedic surgery in a COVID-19-free ‘green’ site. It includes the morbidity and mortality outcomes of those patients who underwent non-emergency orthopaedic operations during this time.

Methods

This is a prospective cohort study over an eight-week period evaluating 104 patients undergoing non-emergency orthopaedic procedures through a COVID-19-free surgical pathway. The primary outcome measure was 14-day postoperative mortality. The main secondary outcome measures were the development of a COVID-19 infection in the hospital and 14 days postoperatively as well as the need for intensive care unit admissions.

Results

No patients developed a COVID-19 infection. There were no intensive care unit admissions or postoperative deaths during our study time frame. There was no statistical difference seen for age (< 70 or > 70), gender, body mass index, or American Society of Anesthesiologists (ASA) grades in the development of postoperative complications.

Conclusions

This study describes a roadmap to setting up a protocolised elective operating service for orthopaedic surgery. It has shown that standardised protocols in a COVID-19-free ‘green’ site, preoperative COVID-19 testing, and adherence to national guidelines on self-isolation can help prevent developing COVID-19 infection postoperatively and reduce the risk of postoperative mortality.

## Introduction

Coronavirus disease 2019 (COVID-19) was declared a pandemic on March 11, 2020 [1]. There were over two million cases and 70,541 deaths in England alone as of January 9, 2021 [2]. The National Health Service (NHS) England and British Orthopaedic Association (BOA) advised postponing all non-emergency elective surgeries beginning April 2020 to reduce avoidable exposure of patients and staff to the virus, increase critical care capacity, and free up staff to manage the surge in COVID-19 patients [3-4].

We initially emerged from the first peak and reached a stage where we could restart non-urgent services, such as elective orthopaedic surgery. Most orthopaedic patients requiring surgery are a vulnerable group at higher risk of contracting COVID-19 in the perioperative phase. A mortality rate of 20.5% (seven of 34) was reported in those who underwent elective surgery in Wuhan City [5] and this was corroborated in a further study by the COVIDSurg Collaborative reporting an overall 30-day mortality rate of 18.9% (53 of 280) in patients who underwent elective surgery [6]. Lazizi et al. described a protocolised operating system in a stand-alone orthopaedic hospital during the pandemic for trauma patients, resulting in only 4% (four of 91) developing COVID-19 after acute emergency trauma surgery [7].

Our acute general hospital was one of the first across England to restart elective orthopaedic surgery and was amongst the first hospitals in the country to carry out elective joint arthroplasty surgery in the midst of the pandemic. Our experiences have taught us that COVID-19 will remain in society for the unforeseeable future. From the lessons we learned, it is more important than ever to have pathways with stringent infection control measures to allow us to continue elective surgery safely to improve the quality of life of people with chronic disabilities.

This study describes the restart of elective orthopaedic surgery after the first peak of COVID-19 in a controlled environment and presents postoperative outcomes of patients who underwent non-emergency orthopaedic operations.

## Materials and methods

Development of a COVID-19-free elective surgical pathway 

Healthcare Provision in the United Kingdom (UK)

Health care in the UK is provided by the NHS which is a publicly funded healthcare system. All UK citizens receive free health care at the point of delivery. Private health care is also available in the UK and provides patients with acute and long-term care services. 

Securing a COVID-19-free Site

NHS England and NHS Improvement purchased extra capacity and support from the independent sector (private care) hospitals [8-9]. As per the BOA’s guidance, our hospital secured a COVID-19-free ‘green’ site with an independent sector hospital to commence elective orthopaedic surgery for NHS patients [10]. 

Infrastructure

Our green site was a separate building site with separate diagnostic facilities. It consisted of 40 single occupancy rooms with ensuite bathrooms. Visiting was suspended. Staffing was provided by the independent sector hospital to minimise cross-contamination between COVID-19 and COVID-19-free sites. Only orthopaedic surgeons and anaesthetists worked across both sites. To limit virus transmission, the staff did not work on both sites on any given day and were screened at the entrance (temperature check and COVID-19 symptom questionnaire). 

Surgical Prioritisation

The Royal College of Surgeons (RCS) of England issued guidance on prioritisation of patients requiring surgery during the pandemic: (1a) emergency: within 24 hours, (1b) urgent: within 72 hours, 2) surgery can be deferred for up to four weeks, (3) surgery can be delayed for up to three months, and (4) surgery can be delayed for more than three months [11]. We used a three-step approach to prioritise patients for elective surgery. Orthopaedic consultants graded each case on their waiting list as per the RCS guide. Patients within each priority level were ranked according to the length of time on the waiting list. These patients were further categorised into low, intermediate, and high-risk based on their American Society of Anaesthesiologists (ASA) physical status and estimated length of stay in the hospital. The higher priority patients were chosen, and those waiting the longest received preference for surgery. 

Self-isolation

National guidance during our study time frame recommended that patients should self-isolate for 14 days prior to elective surgery [12] to reduce the effects of surgical stress during the incubation period [5]. We created a self-isolation guide (Appendix A-1) and a COVID-19 supplementary consent (Appendix A-2) which describe the COVID-19-related risks. These were sent to patients prior to surgery. 

Preoperative Assessment

**Figure 1:**
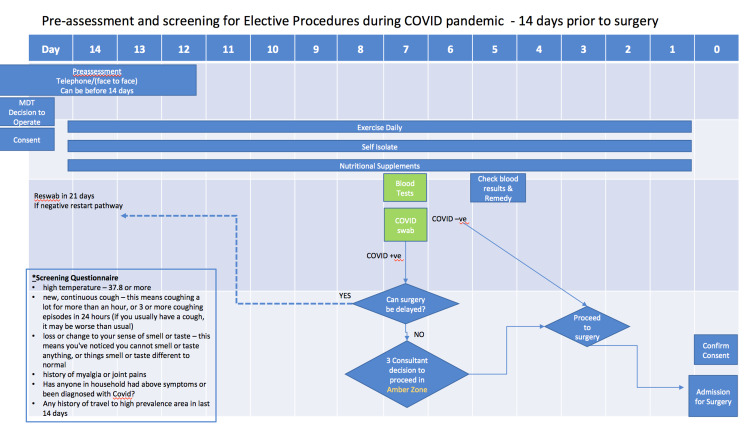
Pre-assessment and screening for elective procedures during COVID pandemic - 14 days prior to surgery MDT: multidisciplinary team; -ve: negative; +ve: positive

Telephone pre-assessment was carried out 15 days prior to surgery, and face-to-face pre-assessment was completed five to seven days before surgery at the COVID-19-free site. Preoperative blood studies, SARs-CoV-2 reverse transcription-polymerase chain reaction (RT-PCR) swab, the COVID-19 screening questionnaire, and the supplementary consent form were completed at this appointment. COVID-19 swab results were available within 48 hours. If the COVID-19 swab was positive, surgery was postponed and the patient was re-swabbed in 21 days (Figure 1). 

Elective Admission Protocol

**Figure 2:**
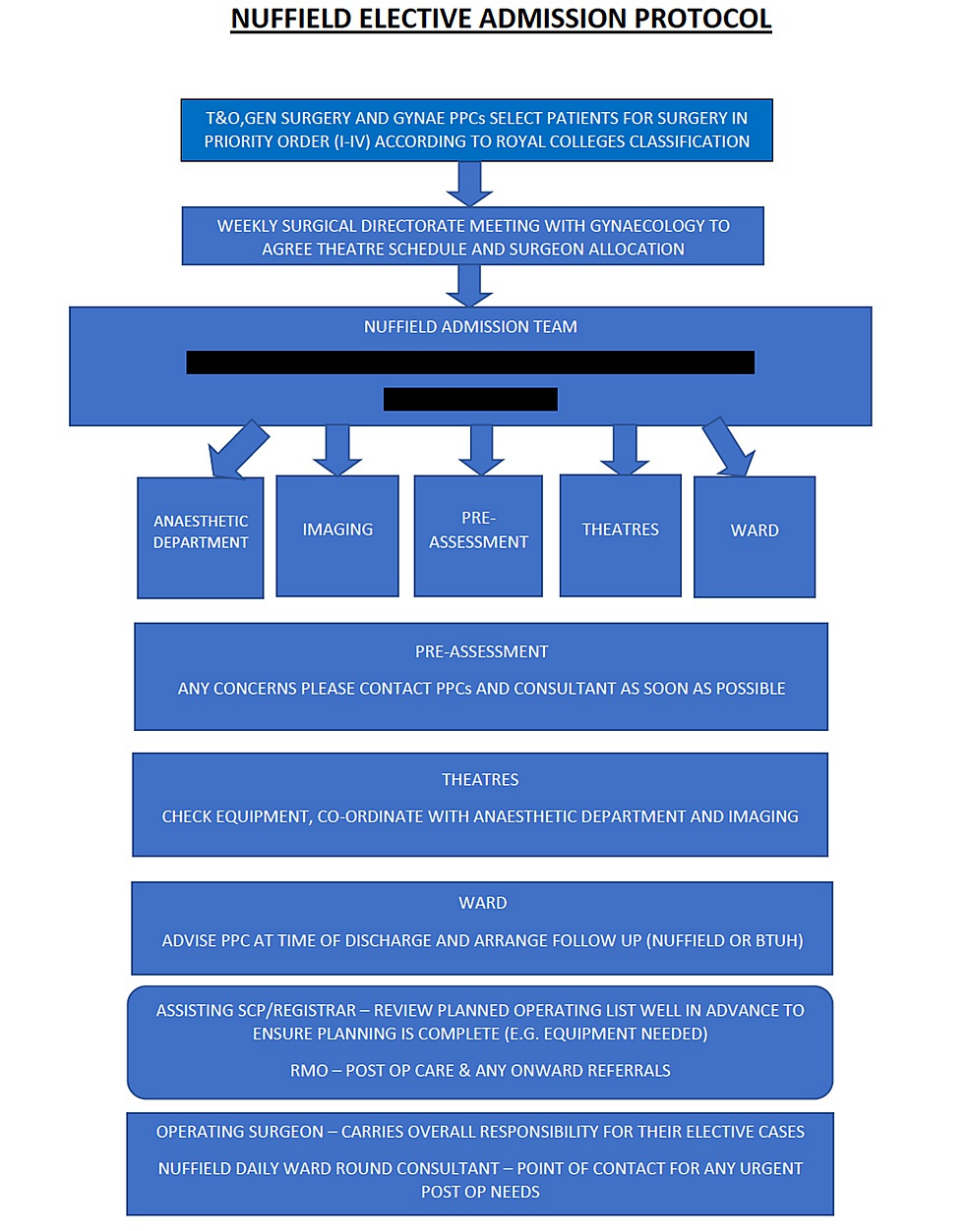
Elective admission protocol BTUH: Basildon and Thurrock University Hospitals; GYNAE: Gynaecology; PPC: patient pathway coordinators; RMO: resident medical officer; SCP: surgical care practitioner; T&O: Trauma and Orthopaedics

Figure 2 describes the pathway leading up to the day of surgery. The surgical team (surgeon, patient pathway coordinators (PPC), and theatre coordinator) reviews the final operating list, ensuring all required equipment, patient notes, results, and images are transferred across to the green site. 

On the day of surgery, patient arrival times were staggered. Patients were met at the entrance of the hospital, self-isolation confirmed, COVID-19 swab results checked, the temperature measured, and a COVID-19 symptom questionnaire completed. If the above were satisfactory, patients were escorted to the ward one at a time. 

Postoperative Care

Daily ward rounds were carried out by the surgical and medical registrar, who were also available to assist with any urgent perioperative needs. They were supported by the independent sector resident medical officer (RMO) who was available 24 hours a day, seven days a week. A junior doctor was allocated for night cover, in addition to the RMO. Patient pathway coordinators organised a virtual telephone consultation six weeks post-surgery and appropriate physiotherapy referrals were made. Wound check and removal of sutures were carried out by the community orthopaedic team who provided care for patients in their own home for up to six weeks from surgery, thus minimising exposure to COVID-19 due to hospital visits. Any concerns were escalated to the operating surgeon. 

Personal Protective Equipment (PPE)

As per Public Health England (PHE) guidance, our staff were required to have eye protection, a fluid repellent surgical gown with a plastic apron underneath, and double gloves for all procedures. In non-aerosol-generating procedures (AGP), staff wore fluid-resistant surgical masks (FRSM), and for AGP, they wore fit-tested filtering facepiece class 3 (FFP3) masks [13]. PPE were changed between cases for the scrubbed team and anaesthetist. The rest of the team members were recommended the sessional use of PPE. 

Patient outcomes data collection 

This was a single centre, prospective cohort study over an eight-week period (May 18 - July 10, 2020) evaluating all patients undergoing non-emergency orthopaedic procedures through the COVID-19-free surgical pathway with a 14-day follow-up postoperatively. Patient demographics, preoperative, operative, and postoperative details were obtained from patient medical records. In view of the secondary use of information, which was collected in the course of normal care, approval from the Research Ethics Committee within the United Kingdom Health Departments Research Ethics Service and Health Research Authority was not deemed necessary. The Charlson’s comorbidity index, which correlates to a predicted 10-year survival rate was used to quantify patient comorbidities [14-16]. 

Elective surgical volume 

The volume of elective orthopaedic surgeries and theatre utilisation during the pandemic at the COVID-19-free site was obtained from the theatre management system for our study time frame. This was compared with the same time period, one year prior, when elective operating was at full capacity at the main hospital site, pre-pandemic. 

Statistical analysis 

GraphPad Prism software (GraphPad Software, San Diego, CA, USA) was used for statistical analysis. A statistically significant level of p < 0.05 was set. Fisher’s exact test was used to assess differences between the development of complications for age (< 70 and > 70) and gender. Chi-squared test was used to assess differences between the development of complications for body mass index (BMI) and ASA grade. 

## Results

One hundred and four patients had elective orthopaedic surgery during our study time frame. The mean age was 59 years (range: 18 to 88). The male to female ratio was 59 (57%):45 (43%). Sixty-eight patients (65%) had a Charlson’s index score of 0 - 2 (10-year survival rate > 90%), 29 patients (28%) scored 3 - 4 (10-year survival rate 53% - 77%), and seven (7%) scored 5 (10-year survival rate 21%).

All patients had a negative RT-PCR swab test, and all patients (except one) adhered to the preoperative 14-day self-isolation protocol. All patients had a recorded temperature of < 37.7° Celsius on the day of admission.

Seventy cases (67.31%) were lower limb, 29 (27.88%) were upper limb, and five (4.81%) were spinal cases. Table 1 summarises the surgical procedures performed and their priority levels as per the RCS surgical prioritisation guidance. Seventy-eight patients (75%) received general anaesthesia and nine (9%) received spinal anaesthesia. These patients had a length of stay ranging from zero to seven days. Seventeen patients (16%) had local anaesthesia with a length of stay ranging from zero to four days (Table 2).

**Table 1:** Procedures at the COVID-19-free ‘Green’ Site, Including Their Priority Levels

Procedure	Number of cases	Priority level
Hip		
Primary total hip replacement (including complex primary cases)	15	3 - 4
Revision total hip replacement	3	3 - 4
Excision of trochanteric bursa	1	3
Knee		
Primary total knee replacement (including complex primary)	12	3 - 4
Revision total knee replacement	3	2 - 4
Aspiration of total knee replacement	1	2
Medial unicompartmental knee replacement	1	4
Knee arthroscopy ± meniscal repair	16	3
Anterior cruciate ligament reconstruction	3	2 - 3
Excision of encapsulated cyst at the back of knee	1	3
Shoulder		
Shoulder arthroscopy + proceed (subacromial decompression, acromioplasty, rotator cuff repair, acromioclavicular joint excision, biceps tenotomy, bursectomy)	9	2
Removal of metalwork (hook plate) from shoulder	1	3
Manipulation under anaesthesia (MUA) shoulder	3	3
Pectoralis major repair	1	2
Foot & Ankle		
Tendo-Achilles reconstruction + excision of ossicle big toe	1	3
Revision ankle non-union to tibiotalocalcaneal fusion	1	3
Metatarsophalangeal joint (MTPJ) fusion + metatarsal osteotomy	4	4
Zadek’s procedure of the big toe	1	4
Scarf + Akin osteotomy of the big toe	3	4
Proximal interphalangeal joint (PIPJ) of the foot	2	4
Removal of metalwork from the foot	2	4
Spine		
Epidural injection	1	2
Spinal decompression ± discectomy	4	2
Hand		
Carpel tunnel decompression	15	3
Total number of cases	104	

**Table 2:** Type of Anaesthesia Received, ASA Grade, and Corresponding Length of Stay ASA: American Society of Anesthesiologists

Anaesthesia	Length of stay in hospital (days)	ASA grade			Total
		1	2	3	
General anaesthesia	< 1	7	16	5	28
	1	4	9	6	19
	2	3	5	-	8
	3	1	8	6	15
	4	2	3	-	5
	5	-	1	-	1
	6	-	-	1	1
	7	-	1	-	1
General anaesthesia total		17	43	18	78
Local anaesthesia	< 1	5	5	4	14
	1	-	1	-	1
	3	-	1	-	1
	4	-	1	-	1
Local anaesthesia total		5	8	4	17
Spinal anaesthesia	< 1	-	2	-	2
	2	-	2	-	2
	3	-	3	-	3
	7	-	1	1	4
Spinal anaesthesia total		-	8	1	9
Total		22	59	23	104

Twenty-two (21.15%) of the 104 patients developed 23 complications whilst in the hospital or during the 14-day postoperative period (Table 3). In those who had complications, 10 (45%) were aged > 70, 14 (64%) were male, eight (36%) were female, the largest cohort for ASA grades was ASA 2 (45%), and the BMI was between 25 - 29.9 (41%). There was no statistical difference found in the development of complications for age (< 70 or > 70) (p = 0.12), gender (p = 0.63), BMI (p = 0.76), or ASA grades (p = 0.24).

**Table 3:** Postoperative Patient Outcomes CTPA: computed tomography pulmonary angiogram; GP: general practitioner; ITU: intensive care unit; IV: intravenous; RT-PCR: reverse transcription-polymerase chain reaction

Patient Outcomes	Management	Number (%) N = 104
Postoperative COVID-19 infection	None	0 (0.00%)
ITU admissions	None	0 (0.00%)
In-hospital deaths	None	0 (0.00%)
Postoperative complications	Management	Number (%) N = 104
Pulmonary embolism	Rivaroxaban for 3 months	2 (1.92%)
Transient ischemic attack	Self-resolved; readmitted for elective carotid endarterectomy	1 (0.96%)
Chest pain	Self-resolved; non-cardiac, non-pulmonary	1 (0.96%)
Fast atrial fibrillation (AF) new diagnosis	Beta blockers. Incidental finding of pheochromocytoma on CTPA requiring readmission to a tertiary centre for management	1 (0.96%)
Postoperative pyrexia	Negative RT-PCR swab and septic screen	3 (2.88%)
Deep joint infection (total knee replacement)	Readmitted for open washout of prosthetic joint	1 (0.96%)
Superficial wound infection post-knee arthroscopy	1 treated with oral antibiotics; 1 readmitted for 24 hour IV antibiotics	2 (1.92%)
Oozy wound	5 treated with pressure dressings; 1 required transfusion of blood products	6 (5.77%)
Symptomatic anaemia	Red blood cell transfusion	4 (3.85%)
Postoperative hypoglycaemia	GP referral for endocrine review	1 (0.96%)
Aborted surgery (irreparable chronic tear of pectoralis major tendon)	Referred to a tertiary centre	1 (0.96%)

No intensive care unit (ITU) admissions or in-hospital deaths occurred during our study period. No patients developed COVID-19 infection in the 14-day postoperative period. In those who had postoperative pyrexia (three patients), the RT-PCR swab test was negative as was the septic screen. Postoperative pyrexia resolved without intervention and was secondary to the stress response of surgery.

Pulmonary complications were experienced by two patients, aged > 80 with an ASA 3. They were diagnosed with a pulmonary embolus following a total knee replacement and a revision of an intramedullary femoral nail to a total hip replacement. They were commenced on rivaroxaban for three months and have made a full recovery.

A male patient (age 65, ASA 2) informed the surgical team after his surgery that he only isolated for 10 days instead of 14 days preoperatively. He developed a superficial infection of the medial portal site after an arthroscopic partial medial meniscectomy, which was treated with oral antibiotics. All patients with postoperative complications have made a full recovery with no further presentations to the hospital.

There were a total of 37 operating days at the COVID-19-free site during our study time frame. During the same time frame one year prior, there were also 37 operating days. Pre-pandemic 2.57 elective theatres on average were allocated for elective procedures per day compared to an average of 1.11 elective theatres at the COVID-19-free site. The total time allocated pre-pandemic was 827.5 hours, of which 80.1% was utilised to complete 380 operations, whereas, at the COVID-19-free site, the total theatre time allocated was 348.5 hours of which only 58.7% was utilised to complete 104 operations.

## Discussion

Discouraging evidence has been available thus far regarding perioperative mortality following elective surgery during the pandemic, with mortality rates of 18.9% and 20.5% in patients who received elective surgery during the pandemic [5-6]. Both studies were published during the first peak when RT-PCR testing was not readily available and there were no standardised protocols or guidance on patient selection/surgical prioritisation, preoperative testing, and preoperative self-isolation.

Many national guidelines have been published on resuming elective surgery in the United Kingdom [3, 8, 10]. We describe our experience of developing a highly stringent protocolised system, based on national guidelines, in a stand-alone COVID-19-free site. One hundred and four adult patients underwent elective orthopaedic surgery.

In eight weeks, one hundred and four adult patients underwent elective orthopaedic surgery through the COVID-19-free surgical pathway we created. Patients were selected according to clinical urgency and were not discriminated against due to age, BMI, ASA, or comorbidities. Despite not excluding higher-risk patients, we had no patients that developed COVID-19 symptoms or had a positive test in the 14 days following surgery. We also had no ITU admissions or postoperative mortality.

A study published by an acute general hospital in the UK evaluated their phased return to elective orthopaedic surgery [17]. They had a similar sample size to our study but excluded patients over the age of 70, BMI > 30, ASA > 2, and those with diabetes, previous stroke, arrhythmias, atrial fibrillation, chronic kidney disease, and hypertension. Their extensive exclusion criteria of high-risk patients resulted in no in-hospital complications, but despite this, one patient tested positive for COVID-19 postoperatively.

The United Kingdom Foot and Ankle COVID-19 National (UK-FAlCoN) study assessed 6,644 adult patients who underwent foot and ankle surgery from 43 centres across the UK during the pandemic in both green and non-green surgical pathways [18]. A total of 35 patients contracted COVID-19: 30 (0.65%) from the non-green pathway versus five (0.26%) from the green pathway. The 30-day COVID-19-related mortality rate was eight (26.67%) in the non-green pathway versus one (20%) in the green pathway. This study suggests that performing surgery in a green pathway reduces the chance of contracting the COVID-19 infection. However, COVID-19-related mortality remains high regardless of which pathway.

Evidence suggests 5% - 80% of those who test positive for COVID-19 may be asymptomatic [19]. A multicentre study found 11% of patients with hip fractures were asymptomatic but tested positive for COVID-19 [20]. Symptom screening alone is insufficient prior to surgery. The RT-PCR swab test has a high specificity (95%) and moderate sensitivity (70%) [21]. Kader et al. predicted a one in 1,400 probability of having COVID-19 infection in patients with a negative RT-PCR test [22].

The World Health Organisation recommended 14 days of isolation prior to having elective surgery during our study time frame. The incubation period for COVID-19 is 10 to 14 days, with one study reporting 97.5% of people with COVID-19 developed symptoms within 11.5 days of exposure [23]. The stress of surgery during the incubation period can exacerbate disease progression, leading to poor outcomes as shown by Lei et al. [5]. The protocols we created ensured that 99% of our patients self-isolated and all had a negative RT-PCR swab test preoperatively. This resulted in no patients developing COVID-19 infection postoperatively or requiring readmission due to COVID-19. The overall 14-day mortality in this study was 0%, and we can confirm that the 30-day mortality remains 0% in this cohort.

Challenges were faced during our transition back to elective orthopaedic work. The independent sector hospital we partnered with had its own in-house information technology system, which did not link to our systems. It proved challenging to access patient electronic medical records and imaging. Current practise involved images being transferred to the COVID-19-free site via the Sectra Image Exchange Portal (Sectra AB, Linköping, Sweden) and a hard copy of patient medical records and clinic letters transferred via hospital transport before surgery. This system involved a high level of timely organisation to allow for the safe management of our patients. Operation notes, postoperative inpatient ward notes, and postoperative images were transferred to be scanned into the electronic system at the main NHS hospital. Even though this wasn’t an issue for us, it is important to consider challenges, such as misplaced patient notes during the transfer from one hospital to another and inaccessibility of notes in time for a follow-up review. Surgical implants were procured beforehand, on occasion requiring transportation from the main hospital, which could result in a delayed arrival or they may arrive de-sterilised, leading to the cancellation of a procedure. We tried to mitigate this by holding implants and equipment at the COVID-19-free site which has its own sterilisation facilities, but this increases the financial implication to our hospital.

We have not been able to revert to the pre-pandemic surgical volume and our theatre utilisation was 22% less, as compared to the same period in 2019. This is likely due to recently restarting a service that has undergone drastic changes to ensure high standards of infection control and safety to patients and staff. Aerosol clearance times after aerosol-generating procedures and regularly changing PPE as per protocol is vital but this means a slower turnover of patients in the operating theatre. With COVID-19 likely to remain within our society for the foreseeable future, these measures will have to continue, but we will become more familiar with the processes and be able to provide a smooth-running service to patients over time. As COVID-19 prevalence reduces and green site working becomes more established, we feel theatre downtime will also progressively reduce.

The authors recognise the limitations that must be acknowledged when interpreting the results of this study. Firstly, postoperative COVID-19 testing was not performed routinely. We understand that there is a high proportion of asymptomatic patients who can spread the disease. However, our patient cohort self-isolated postoperatively and remained asymptomatic. Secondly, this study only accounts for eight weeks of operating with short postoperative follow-up. The generalisability of our patient outcomes to other centres is unknown and should be interpreted with caution. Local disease prevalence can also have an impact on the reproducibility of the results.

Further research with larger sample size and longer follow-up is necessary to assess the long-term effects. However, this study aimed to describe the process of safely restarting elective orthopaedic surgery and present initial patient outcomes which are reassuring and validate our COVID-19-free surgical pathway.

We need to learn to co-exist with COVID-19 in our society and be able to provide the required care for our patients. This study describes a roadmap to setting up a protocolised elective operating service for orthopaedic surgery, providing guidance to centres on how to develop protocols and overcome challenges to resume elective surgery safely. This is a preliminary study but has shown a safe restart of elective orthopaedic surgery in the midst of a pandemic with positive patient outcomes.

## Conclusions

This study describes a roadmap to setting up a protocolised elective operating service for orthopaedic surgery. It has shown that standardised protocols in a COVID-19-free ‘green’ site, preoperative COVID-19 testing, and adherence to national guidelines on self-isolation can help prevent developing COVID-19 infection postoperatively.

## Appendices

APPENDIX A-1: Self-Isolation Guide

SELF-ISOLATION PRIOR TO SURGERY AT BRENTWOOD NUFFIELD HOSPITAL

- If you do not self-isolate, then you are risking your own health and the health of others!

- Staying at home for 14 days before your forthcoming surgery is essential as it will greatly reduce the chance of catching an infection from the community while you are awaiting surgery. If you are having major surgery, such as joint replacement, spinal surgery, implant surgery, major abdominal/gynaecological surgery, cancer surgery, or surgery involving prolonged anaesthetic, the current medical advice is that you also self-isolate for 14 days postoperatively.

- If you cannot move other vulnerable people out of your home, stay away from them as much as possible.

- Reduce the spread of infection in your home: wash your hands regularly for 20 seconds, each time using soap and water, or use hand sanitiser.

- If you develop coronavirus (COVID-19) symptoms (flu-like symptoms, cough, fever, sore throat, muscle aches, loss of sense of smell), please let us know immediately by calling Brentwood Nuffield on 01277 695 695 and asking for the Pre-Assessment Service.

- Only go outside for any urgent health reasons - please contact NHS 111 for advice (telephone or online).

- When traveling to the hospital for your pre-assessment appointment and on the day of surgery, please travel alone or with a member of your household. You are permitted to use a taxi service to/from Brentwood Nuffield but you should wear a face covering and gloves.

- Wash your hands as soon as you get home.

- Do not meet others, even friends or family.

- You and all household members should remain at home. Do not go to work or public areas, and do not use public transport. If family members need to go out to work, consider alternative accommodation for them during your period of self-isolation, or if that is not possible, consider isolating them in their rooms.

- You should not go out, even to buy food or other essentials, and any exercise should be taken within your home.

- Both before and after your procedure, please plan ahead. If you require help with buying groceries, other shopping, picking up medication, or walking a dog, you will need to ask friends or relatives to help. Alternatively, you can order medication by phone or online. You should consider ordering your shopping online. Make sure you tell delivery drivers to leave items outside. The delivery driver should not come into your home.

- Do not invite or allow social visitors, such as other friends and family, to enter your home. If you want to speak to someone who is not a member of your household, use the phone or social media.

- Seek prompt medical attention if your illness or the illness in any household members is worsening. If it’s not an emergency, contact NHS 111 online. If you have no internet access, you should call NHS 111. If it is an emergency and you need to call an ambulance, dial 999.

- All routine medical and dental appointments should usually be canceled while you and the family are staying at home.

TIPS FOR SELF-ISOLATION

Staying at home may be difficult and frustrating, but there are things that you can do to help make it easier. These include:

- plan ahead and think about what you will need in order to be able to stay at home,

- talk to your employer, friends, and family to ask for their help to access the things you will need to make your stay at home a success,

- think about and plan how you can get access to food and other supplies such as medications that you will need during this period,

- ask friends or family to drop off anything you need or order supplies online, but make sure these are left outside your home for you to collect,

- make arrangements for someone to walk your dog during your isolation period,

- make sure that you keep in touch with friends and family over the phone or through social media,

- think about things you can do during your time at home. People who have successfully completed a period of staying at home have kept themselves busy with activities, such as cooking, reading, online learning, and watching films,

- many people find it helpful to plan out the full self-isolation time on a calendar. You may also find it helpful to plan in advance what you will do if, for example, someone in the household were to feel ill.

- If you follow these guidelines, we are confident that you will have done your best to ensure a good outcome from your forthcoming surgery.

Date:

APPENDIX A-2: COVID-19 Supplementary Consent Form

SURGICAL CARE DURING THE CORONAVIRUS (COVID-19) PANDEMIC

Patient’s surname/family name: ………………………………………….....................

Patient’s first names: ………………………………………….........................................

Date of birth: ………………………………………….......................................................

Health professional seeking consent: …………………………………………..........

Job title: …………………………………………................................................................

NHS number (or other identifiers): ………………………………………….................

Patient record of discussion and awareness of issues: ...........................

Statement of health professional seeking consent: ..................................

KEY INFORMATION FOR PATIENTS:

The Mid and South Essex University Hospitals will do everything we can to provide essential surgery at this time. However, the coronavirus pandemic is placing huge demands on the entire health service and creating new challenges. This form is to make you aware that your surgical care may be affected in many ways.

We must be clear that:

- Your assessment and care may be disrupted, delayed, or performed differently during the pandemic.

- Coming to the hospital might increase your chances of contracting the COVID-19 virus, or you may be already carrying it when you come for your operation.

- If the coronavirus infection occurs when you have surgery or whilst in the hospital, this could make your recovery more difficult or increase your risk of serious illness or death.

- We will do everything we can to perform your operation, keep you safe, and provide you with information at all stages. We will listen to your concerns and discuss them.

- We will try to minimize face-to-face contact before and after your operation. As a result, we may assess you by telephone consultations, and share postoperative results by telephone or letter.

- You may wish to delay your operation, and we would understand your reasons for this. However, future dates for surgery may take much longer than normal to arrange.

These are examples of the ways in which your surgical care may be different from normal:

Before your operation

- Most of your consultations will occur by telephone or by email and letter.

- We will rely on your local hospital to send important test results and letters to us.

- We may also ask you to email or post medical information to us.

- You will be sent a copy of your consent form that explains your operation.

- You may be sent preoperative information by post or email to explain your operation and potential complications.

- Your anaesthetic assessment will be by telephone with a nurse, and possibly an anaesthetist, too.

- We may arrange for you to have coronavirus testing before your operation.

- Your operation would be likely to be postponed if you test positive or are unwell.

- Routinely, we will ask you to go into strict isolation before a procedure unless it is an emergency. You will be given clear information about this.

Your operation

- Your operation may not take place on the site that you were expecting. We will be operating at NHS sites across Essex and also at the local private hospitals, including the Nuffield Hospital in Brentwood, Springfield in Chelmsford, and the Wellesley in Southend.

- Circumstances will be very different in the hospital. Wards will be reorganised, and staff will be wearing protective equipment.

- You may not meet your surgeons until the day of treatment, and they might not be the surgeon you expected. They will, however, be experienced and trained to perform your operation.

After your operation

- You will be discharged from the hospital as soon as you are ready, or may be moved elsewhere to a ‘step-down’ unit to complete your recovery.

- We will check on you by telephone.

- Some follow-up care or emergency admission may need to happen at your local hospital.

- It is likely you will not be able to have your family and friends visit whilst in the hospital.

- You may be able to contact your family using social media or video calls.

Name and Signature of the responsible clinician

Signed: …….…………………………………… Date ……....……………………………………..

Name (PRINT) ………………………..………................................................................

Job title ………………………………………….....................……………………………………..

Statement of an interpreter (where appropriate)

I have interpreted the information above to the patient to the best of my ability and in a way in which I believe he/she can understand.

Signed ……………….……………………………….      Date…….………………………………

Name (PRINT) …………….…………………………………………………………………………..

**Statement of the patient**

I acknowledge the information above, which I have read, together with my surgical consent form.

Signature ……………………………………….................................

Date ……………………………..………………..................................

Name (PRINT) ……………………………………………………………

Relationship to patient ………………………….......................

Confirmation of consent

(to be completed by a health professional when the patient is admitted for the procedure if the patient/parent has signed the form in advance)

I have confirmed that the patient has no further questions and wishes the procedure to go ahead.

Signed: ……………………………………………...............................

Date ……....…………………………….............................................

Name (PRINT) ………………………..………................................

Job title …………………………………..........................................
